# Phase 1 Studies of the Anti-Tau Monoclonal Antibody JNJ-63733657 in Healthy Participants and Participants with Alzheimer’s Disease

**DOI:** 10.14283/jpad.2024.163

**Published:** 2024-09-10

**Authors:** Wendy R. Galpern, G. Triana-Baltzer, L. Li, K. Van Kolen, M. Timmers, K. Haeverans, L. Janssens, H. Kolb, P. Nandy, K. Aida, H. Shimizu, M. Mercken, H. Sun

**Affiliations:** 1 Janssen Research & Development, LLC, 1125 Trenton-Harbourton Road, Titusville, NJ 08560 USA; 2 Janssen Research & Development, LLC, La Jolla, CA USA; 3 Janssen Pharmaceutica NV, Beerse, Belgium; 4 Janssen Research & Development, LLC, Raritan, NJ USA; 5 Janssen Pharmaceutical K.K., Tokyo, Japan; 6 Present Address: Current affiliation- CSL Behring, King of Prussia, PA USA; 7 Present Address: Current affiliation - Enigma Biomedical Group, Knoxville, TN USA

**Keywords:** Alzheimer’s disease, tau, p217+tau, Phase 1 trial, monoclonal antibody

## Abstract

**Background:**

JNJ-63733657 (posdinemab) is a humanized IgG1/kappa monoclonal anti-phospho tau antibody that binds with high affinity to phosphorylated amino acid 217 (pT217) in the proline-rich domain. The parent molecule, PT3, was raised against Alzheimer’s disease brain purified paired helical filament, and preclinical studies with the humanized version, JNJ-63733657, have demonstrated reductions in tau seeding. The results of the first-in-human clinical trial of JNJ-63733657 and a separate single ascending dose study in Japanese participants are presented.

**Objectives:**

To evaluate the safety and tolerability, pharmacokinetics, immunogenicity, and pharmacodynamics of JNJ-63733657 after single and multiple intravenous dose administrations in healthy participants and participants with prodromal or mild Alzheimer’s disease.

**Design:**

A two part first-in-human, phase 1, randomized, double-blind, placebo-controlled trial: Single ascending dose (Part 1) and multiple ascending dose (Part 2). And a phase 1, randomized, double-blind, placebo-controlled single ascending dose trial in healthy Japanese participants.

**Setting:**

7 sites in Belgium, Netherlands, Spain, and Germany; 1 site in Japan.

**Participants:**

A total of 40 healthy participants aged 55–75 were enrolled in Part 1 of the first-in-human study; a total of 16 healthy participants and 13 participants with prodromal or mild AD aged 55–80 years were enrolled in Part 2. In the Japanese trial, a total of 24 participants aged 55–75 were enrolled.

**Intervention:**

In Part 1, single doses of 1, 3, 10, 30, or 60 mg/kg of JNJ-63733657 or placebo were administered to healthy participants. In Part 2, two dose levels of JNJ-63733657 (5 mg/kg or 50 mg/kg) or placebo were evaluated in healthy participants, and 2 dose levels (15 mg/kg or 30 mg/kg) or placebo were evaluated in participants with Alzheimer’s disease; doses were administered on Days 1, 29, and 57. In the Japanese trial, single doses of 3, 15, or 60 mg/kg of JNJ-63733675 or placebo were administered. All doses were administered intravenously.

**Measurements:**

Safety assessments, serum and cerebrospinal fluid pharmacokinetic parameters, immunogenicity, and cerebrospinal fluid pharmacodynamic changes in free and total p217+tau, total tau, and p181tau were evaluated.

**Results:**

JNJ-63733657 was generally safe and well-tolerated in healthy participants and participants with Alzheimer’s disease. In healthy participants and participants with Alzheimer’s disease, JNJ-63733657 demonstrated linear PK, and serum C_max_ and AUC were approximately dose proportional following single and multiple doses. Dose-dependent reductions in free and total p217+tau in cerebrospinal fluid were observed. No changes in total tau or p181tau were observed in healthy participants whereas Alzheimer’s disease participants showed decreases in these tau species following administration of JNJ-63733657.

**Conclusion:**

In these Phase 1 trials, no safety or tolerability concerns were identified, and dose dependent reductions in p217+tau in the cerebrospinal fluid were demonstrated following administration of JNJ-63733657. The safety and biomarker profiles support the continued investigation of this compound for the slowing of disease progression in Alzheimer’s disease.

**Electronic Supplementary Material:**

Supplementary material is available for this article at 10.14283/jpad.2024.163 and is accessible for authorized users.

## Introduction

**T**he therapeutic landscape for Alzheimer’s disease (AD) is evolving rapidly with anti-amyloid antibodies becoming available for the treatment of early AD (1) and trials ongoing in the preclinical stage (2, 3). While amyloid targeted therapies may offer a significant advance in the management of AD, the slowing of disease progression is incomplete, and the adverse event profile is not benign (1, 4, 5). In addition to extracellular beta-amyloid plaques targeted by these therapies, pathologic tau remains a potential therapeutic target for slowing the progression of AD.

The tauopathy of AD is characterized by aberrant hyperphosphorylation, which interferes with microtubule interactions and tau proteasomal degradation, and also promotes tau misfolding and aggregation. Consequently, paired helical filaments (PHFs) and neurofibrillary tangles (NFTs) develop intracellularly (6). Cortical NFTs correlate with the cognitive decline of AD, even more closely than amyloid beta plaques (7–9), highlighting the rationale for developing tau-targeted therapies for slowing the progression of AD.

Although tau can be released in the extracellular space from injured neurons (10), active secretion also has been reported(11). As reviewed elsewhere, (12) both physiological and pathological tau species have been proposed to be secreted via different mechanisms. In the context of AD, the release of phosphorylated tau (p-tau) is enhanced and positively correlated with the presence of amyloid plaques (13). Upon release, tau exists as single molecule fragments or as aggregates – known as tau seeds – that can be derived from de novo formed oligomers or from existing fibrillary structures like PHFs (14–16) and are hypothesized to propagate between cells in a prion-like manner (6, 17, 18). Tau seeding is observed in extracts derived from postmortem human AD brain and increases with Braak stages (19). Using a highly sensitive FRET-based assay (19), tau seeding activity has been detected in some brain regions prior to biochemical or histological detection of NFTs suggesting that preventing seeding could impact NFT formation and disease progression. Additionally, the spread of tau observed on PET imaging may occur along functionally connected pathways, adding support to the hypothesis that early spatiotemporal progression of NFT across the brain occurs via propagation between neurons and that intercepting pTau seeds at these junctions may attenuate progression of NFT load (20). These data support the hypothesis of early extracellular propagation of tau seeds that precedes development of NFTs (19). Thus, targeting tau seeds with interventions such as monoclonal antibodies may be a promising approach for slowing disease progression in AD.

JNJ-63733657 is a humanized IgG1/kappa monoclonal anti-tau antibody that binds with high affinity to PHF tau and very weakly to non-pTau (21). The parent molecule of JNJ-63733657, PT3, was raised against AD brain purified PHF, and preclinical studies with the humanized version, JNJ-63733657, have demonstrated reductions in tau seeding. The core epitope of tau recognized by JNJ-63733657 contains phosphorylated amino acid 217, and binding is enhanced when neighboring residues (e.g., 212) are phosphorylated, and is thus referred to as “p217+tau”. The mid-domain region where JNJ-63733657 binds differs from that of other anti-tau monoclonal antibodies that target the N-terminus. In Phase 2 trials in AD, these latter antibodies did not demonstrate clinical benefit (22, 23). The N-terminus region of tau from post-mortem AD-derived tau seeds is commonly reduced, likely due to cleavage by proteases. This cleavage leaves less tau containing the N-terminus available for the antibodies to bind and suggests that antibodies binding in the proline rich domain may intercept more tau forms (24). Indeed, although structural biology studies have demonstrated that N-terminus epitopes are highly accessible in PHFs (25), antibodies targeting such epitopes show only limited effects in preclinical models of tau seeding (26). In models using postmortem human brain-derived PHFs, antibodies targeting a more central epitope have more pronounced effects on seeding (26). Accordingly, JNJ-63733657 has demonstrated a reduction of tau seeding in an in vitro cellular depletion study while the murineversion of this antibody has shown reduction in tau pathology in animal models of tau seeding following peripheral injection (21).

A sensitive assay is available that can quantify p217+tau fragments in cerebrospinal fluid (CSF) enabling evaluation of the pharmacodynamic (PD) response of JNJ-63733657 (27). Following administration of JNJ-63733657, changes in levels of JNJ-63733657 bound to p217+tau (monomeric tau) in the CSF can be quantified and indicate that antibody is binding to tau species that contain the p217 epitope in the CSF and presumably also in the interstitial fluid (ISF) in the brain.

Here we report the results of the phase 1 trials evaluating the safety and tolerability, pharmacokinetics, immunogenicity, and pharmacodynamics of the high affinity anti-tau monoclonal antibody JNJ-63733657 in healthy participants (non-Japanese and Japanese) and participants with prodromal or mild AD.

## Methods

Two trials are presented: A first-in-human (FIH) phase 1, randomized, double-blind, placebo-controlled trial conducted in 2 parts: Part 1 evaluated single ascending dose (SAD) levels of JNJ-63733657 in healthy participants, and Part 2 evaluated multiple ascending dose (MAD) levels in healthy participants and participants with prodromal or mild AD. This study was conducted from 04 January 2018 to 16 December 2019 at 7 sites in Belgium, Netherlands, Spain, and Germany. The second trial was a phase 1, randomized, double-blind, placebo-controlled, SAD trial in healthy Japanese participants conducted from 01 October 2018 to 11 July 2019 at 1 site in Japan.

### Ethics

Study protocols and amendments were reviewed and approved by local ethics committees. The trials were conducted in accordance with the ethical principles that were in accordance with the ethical standards of the responsible committee on human experimentation (institutional and national) and with the Helsinki Declaration of 1975, as revised in 2000 and applicable regulatory requirements. Before study participation, written informed consent was obtained from all participants and from study partners of AD participants.

### Study objectives

The primary objective was to assess the safety and tolerability of the anti-tau monoclonal antibody JNJ-63733657 following single IV ascending dose administration in healthy participants and multiple IV ascending dose administration in healthy participants and participants with AD. Secondary objectives were to investigate the PK (pharmacokinetics) of JNJ-63733657 in serum and CSF as well as immunogenicity in serum. Additionally, the effects of JNJ-63733657 on CSF levels of total and free tau fragments containing the p217+ epitope were measured. Changes in CSF levels of total tau (tTau) and p181tau also were explored.

### Study design and treatment

Two trials are presented: A 2-part (SAD and MAD) FIH trial with 9 cohorts and a Japanese SAD trial. For all participants, the studies consisted of 3 phases: A screening phase (8 weeks), a double-blind treatment phase (13 weeks for SAD, 21 weeks for MAD), and a follow-up phase (2 weeks). The total study duration was approximately 23 weeks in the SAD and 31 weeks in the MAD.

In the SAD part of the FIH study, sequential cohorts of 8 healthy participants received JNJ-63733657 at dose levels of 1, 3, 10, 30, or 60 mg/kg IV or placebo with a randomization ratio of 3:1 (JNJ-63733657:placebo). In the MAD part, 2 dose levels of JNJ-63733657 (5 mg/kg or 50 mg/kg) or placebo were evaluated in cohorts of 8 healthy participants per cohort, and 2 dose levels (15 mg/kg or 30 mg/kg) or placebo were evaluated in up to 8 participants per cohort with prodromal or mild AD. Dosing took place on Days 1, 29, and 57 with a 3:1 JNJ-63733657:placebo randomization ratio. In the Japanese SAD trial, sequential cohorts of 8 healthy participants received JNJ-63733657 at dose levels of 3, 15, or 60 mg/kg IV or placebo with a 3:1 JNJ-63733657:placebo randomization ratio.

Sentinel dosing was undertaken in the SAD part of the FIH study. If 2 or more participants were available for dosing at the initiation of any given MAD cohort, then sentinel dosing was done. As the dose levels had been evaluated previously in the FIH study, sentinel dosing was not included in the Japan study. During their first IV administration, participants for the SAD part of the study had a 5-day/4-night inpatient period, and participants for the MAD part of the study had a 3 day/2-night inpatient period. On subsequent dosing days for the MAD part, participants came to the unit on the day of IV administration and were discharged at least 1 hour post IV infusion. Participants in the Japan study also had a 5-day/4-night inpatient period. For the SAD studies, participants returned to the site for follow-up visits up to 13 weeks following dose administration. For the MAD part, participants returned to the site for follow-up visits up to 13 weeks following the final dose.

Prior to dosage escalations in the SAD and MAD parts and in the Japan study, the observed safety and tolerability profiles as well as available PK of prior cohorts were reviewed during blinded data visualization meetings. An independent data monitoring committee was not utilized.

### Eligibility criteria

For the FIH study, the SAD part enrolled healthy male and female participants 55 to 75 years old. The MAD part enrolled male and female participants 55 to 80 years old who were healthy or had prodromal or mild AD. Women were not of child-bearing potential. Prodromal or mild AD was defined as having a Clinical Dementia Rating (CDR) Global Score of 0.5 or 1.0 and evidence of amyloid deposition and tauopathy as demonstrated by an abnormal CSF Aβ_1–42_ (< 600 pg/mL) and Aβ_42/40_ ratio (< 0.89), and elevated CSF p181tau (> 70 pg/mL) or tTau (> 350 pg/mL). Participants with AD were required to have a reliable study partner. Additional eligibility criteria included body mass index of 18–35 mg/m^2^, and healthy participants were required to have an MMSE score > 27 at screening. Participants were excluded if there was a history of or current liver or renal insufficiency or clinically significant medical, psychiatric, or neurological disorders aside from AD for the MAD part of the study. Use of cholinesterase inhibitors and memantine at dosages stable for at least 6 weeks was allowed for AD participants. Eligibility criteria were similar for the FIH and Japanese SAD studies.

### Study endpoints

#### Safety and tolerability

Safety and tolerability were evaluated throughout the studies by monitoring adverse events, vital signs, physical and neurological examinations, electrocardiograms, and laboratory evaluations including hematology, serum chemistry, and coagulation panels as well as urinalysis. Brain MRIs were evaluated for change from baseline in the FIH study. Telemetry was evaluated on Day 1 from 1 hour before to 12 hours after in the SAD studies.

#### Pharmacokinetic and immunogenicity evaluation

Serum and CSF concentrations of JNJ-63733657 were determined using assays that were validated in samples from healthy participants and participants with AD. The lowest limit of quantification (LLOQ) of serum and CSF are 0.40000 µg/mL and 0.50000 ng/mL, respectively. The anti-drug antibody (ADA) assay was validated in samples from healthy participants and participants with AD. The minimal dilution of the ADA assay is 1/22.5.

Serum samples were collected predose and up to Day 92 in the SAD studies and up to Day 148 in the MAD part. CSF samples (12 mL) were collected at baseline and at a maximum of 4 post-dose timepoints separated by at least 12 days. CSF sampling schemes varied by cohort and were balanced across treatment groups.

Data for all participants receiving at least one dose of JNJ-63733657 were included in the PK analysis. The PK parameters calculated using non compartmental analysis (NCA) following single or multiple IV doses included: C_max_, T _max_, AUC_lastt_, AUG_inf_, AUC_τ_ (MAD only), accumulation ratio (MAD only), CL, and t_1/2_. For calculation of individual pharmacokinetic parameters, serum and CSF concentrations below the LLOQ were treated as being 0 in case of occurrence before the first or after the last measurable serum concentration. Participants were excluded from the PK analysis if their data did not allow for accurate assessment of the PK (e.g., incomplete administration of study drug, missing dosing information, concentration data not sufficient for PK parameter calculation).

Immunogenicity was evaluated by determining the incidence of serum ADA for all participants who received at least 1 dose of JNJ-63733657 and had at least 1 sample obtained after the first dose. Treatment-emergent ADA was defined as participants with increased ADA titer post-treatment compared to baseline. Graphical exploration was performed for serum concentration-time profiles in ADA positive participants versus ADA negative participants in the same cohort.

#### Biomarker and pharmacodynamic evaluation

For AD participant eligibility, screening/baseline CSF samples were analyzed for Aβ_1–42_, tTau, and p181tau using Fujirebio Innotest assays (cat#s 81583, 81579, and 81581, respectively) and Aβ_42/40_ ratio using MSD V-plex assay (cat# K15200E).

PD effects of JNJ-63733657 were assessed in CSF via measurements of free p217+tau and total p217+tau to allow determination of p217+tau bound to JNJ-63733657 (Supplementary Figure 1A–C). The free form is the p217+tau not bound by JNJ-63733657, while the total is both bound and unbound p217+tau. The p217+tau bound to JNJ-63733657 is calculated as the difference between total p217+tau and free p217+tau and serves as an indicator of target engagement. CSF was treated without or with heat denaturing for the evaluation of free or total p217+tau, respectively. Heat denaturing dissociates Tau/JNJ-63733657 complexes and denatures JNJ-63733657. Samples were tested using proprietary, highly sensitive, and epitope-specific assays on a Simoa platform to quantify CSF p217+tau (total and free form) (27). While JNJ-63733657 interferes with the CSF p217+tau assay (Supplementary Figures 1A–C), it does not interfere with measurements of tTau and p181tau therefore heat denaturing was not performed for these latter measures.

To confirm specificity of JNJ-63733657 for the p217+tau epitope, the same samples were analyzed for tTau using an assay that measures a phosphorylation-independent epitope in a similar region of tau (using HT7 and pT82 antibodies) (27) and the p181tau epitope (Fujirebio Innotest cat#81581].

### Statistical analyses

Given the descriptive nature of these Phase 1 studies, no formal statistical hypothesis testing was planned. The number of participants per cohort was selected to allow for the clinical judgment of safety and tolerability and assessment of the PK profile.

For safety assessments, results were summarized for all participants who received at least one dose of JNJ-63733657 or placebo.

For all participants who received at least one dose of JNJ-63733657, descriptive statistics including arithmetic mean, SD, CV, median, minimum, and maximum were calculated for all individual derived PK parameters when appropriate. Descriptive statistics were used to summarize JNJ-63733657 serum and CSF concentrations at each sampling time point for each dose. Immunogenicity results were summarized for all participants who received at least 1 dose of JNJ-63733657 and had at least 1 sample obtained after the first dose of JNJ-63733657. Treatment-emergent ADA was defined as an increase in ADA titer post-treatment compared to baseline.

Descriptive statistics were used to summarize CSF p217+tau (total, free, and bound to JNJ-63733657), tTau, and p181tau at each sampling time point for each dose group. These statistics included absolute concentration, percent changes from baseline for individual participants, and group mean and corresponding 95% confidence intervals (CI). Dependence between baseline CSF p217+tau and percent change in CSF p217+tau and dose-response relationships were explored.

## Results

### Study population and disposition

Demographic and baseline characteristics of all participants who received at least 1 dose of study intervention were generally balanced across the treatment groups (Table 1).

**Table 1:** Demographic and baseline and characteristics for FIH study and Japanese study

**FIH single dose**						
	**JNJ-63733657**					
	**Placebo**	**1 mg/kg**	**3 mg/kg**	**10 mg/kg**	**30 mg/kg**	**60 mg/kg**
n	10	6	6	6	6	6
Age (Years)						
Mean (SD)	65.0 (5.33)	63.8 (3.31)	69.5 (3.89)	61.5 (3.27)	63.5 (6.22)	60.3 (3.67)
Gender						
Male	4 (40.0%)	4 (66.7%)	5 (83.3%)	3 (50.0%)	3 (50.0%)	1 (16.7%)
Race						
White	10 (100.0%)	6 (100.0%)	6 (100.0%)	6 (100.0%)	6 (100.0%)	6 (100.0%)
Ethnicity						
Hispanic or Latino	1 (10.0%)	0	0	0	0	0
Not Hispanic or Latino	9 (90.0%)	6 (100.0%)	6 (100.0%)	6 (100.0%)	6 (100.0%)	6 (100.0%)

**FIH multiple dose**						
			**JNJ-63733657**			
	**Placebo**					
	**AD**	**HP**	**HP**	**AD**	**AD**	**HP**
	**Placebo**	**Placebo**	**5 mg/kg**	**15 mg/kg**	**30 mg/kg**	**50 mg/kg**
n	2	4	6	6	5	5
Age (Years)						
Mean (SD)	65.5 (9.19)	72.8 (3.20)	67.2 (7.55)	71.8 (3.19)	71.4 (4.39)	73.3 (5.65)
Gender						
Male	2 (100.0%)	3 (75.0%)	4 (66.7%)	3 (50.0%)	2 (40.0%)	3 (50.0%)
Race						
Asian	0	0	0	0	0	1 (16.7%)
White	2 (100.0%)	4 (100.0%)	6 (100.0%)	6 (100.0%)	5 (100.0%)	5 (83.3%)
Ethnicity						
Not Hispanic or Latino	2 (100.0%)	4 (100.0%)	6 (100.0%)	6 (100.0%)	5 (100.0%)	6 (100.0%)

**Japanese single dose**				
		**JNJ-63733657**		
	**Placebo**	**3 mg/kg**	**15 mg/kg**	**60 mg/kg**
n	6	6	6	6
Age (Years)				
Mean (SD)	63.7 (4.59)	66.5 (5.96)	65.3 (5.85)	60.3 (2.94)
Gender				
Male	4 (66.7%)	2 (33.3%)	3 (50.0%)	3 (50.0%)
Race				
Asian	6 (100.0%)	6 (100.0%)	6 (100.0%)	6 (100.0%)
Ethnicity				
Not Hispanic or Latino	6 (100.0%)	6 (100.0%)	6 (100.0%)	6 (100.0%)

Footnote: AD, Alzheimer’s Disease; FIH, first-in-human; HP, Healthy participants; SD, standard deviation

In the SAD part of the FIH study, 40 healthy participants (30 randomized to JNJ-63733657, 10 randomized to placebo) were enrolled and received at least 1 dose of study intervention. One participant withdrew from the study for reasons unrelated to safety (not available for follow-up) and 39 participants completed the study. In the MAD part, 29 participants (23 randomized to JNJ-63733657, 6 randomized to placebo) were enrolled and received at least 1 dose of study intervention; 16 were healthy participants and 13 had prodromal or mild AD. Of the 29 participants, 1 AD participant from the 30 mg/kg cohort withdrew from the study for reasons unrelated to safety (declined to undergo additional procedures), and 28 participants completed the study. Of the 40 healthy participants, 39 fit the biochemical criteria for non-AD, and all the AD participants fit the biochemical criteria for AD. In the Japanese SAD study, 24 healthy participants (18 randomized to JNJ-63733657, 6 randomized to placebo) were enrolled and received at least 1 dose of study intervention. All participants completed the Japanese study. Each cohort in the FIH study and the Japanese study enrolled 8 participants (6 active: 2 placebo) except for the 30 mg/kg AD cohort which randomized 5 participants (all active) due to recruitment challenges.

### Safety and tolerability

In the FIH study, no serious adverse events were reported in participants receiving single doses of JNJ-63733657 (1mg/kg–60 mg/kg) in Part 1 of the study. One placebo-treated participant in the SAD part of the study experienced 2 SAEs (post-LP [lumbar puncture] syndrome/suspected post-spinal headache and hypertension). In Part 2, one AD participant treated with JNJ-63733657 15 mg/kg had an SAE of renal neoplasm, which was considered not related to study drug. No clinically important abnormalities were observed in vital sign parameters, laboratory values, or brain MRIs. There were no deaths or early terminations due to treatment-emergent adverse events (TEAEs).

In Part 1, 24 (80%) of the 30 participants treated with JNJ-63733657 reported 1 or more AEs: 50% of participants treated with 1 mg/kg, 66.7% of participants treated with 3 mg/kg, 100% of participants treated with 10 mg/kg, 83.3% of participants treated with 30 mg/kg, and 100% of participants treated with 60 mg/kg. Of the 10 participants treated with placebo, 8 (80%) reported 1 or more AEs.

The most commonly reported TEAE (reported by >20% participants) by preferred term in participants who received JNJ-63733657 1 mg/kg was post-LP syndrome (50%). No TEAEs were reported in more than 1 participant who received JNJ-63733657 3 mg/kg. For participants who received JNJ-63733657 10 mg/kg, the most common TEAEs were post-LP syndrome (83.3%), hypercholesterolemia (50%), nausea (50%), hot flush (50%), vomiting (33.3%), and headache (33.3%), and for participants who received JNJ-63733657 30 mg/kg, hepatic enzyme increase (33.3%). For participants who received JNJ-63733657 60 mg/kg, the most common TEAEs were headache (83.3%), hypercholesterolemia (50%), post-LP syndrome (33.3%), procedural pain (33.3%), muscle spasms (33.3%), and neck pain (33.3%). The most commonly reported TEAEs in participants who received placebo were headache (30%) and back pain (30%). (Table 2A).

**Table 2:** Adverse events

**A. FIH single dose**						
	**JNJ-63733657**					
	**Placebo**	**1 mg/kg**	**3 mg/kg**	**10 mg/kg**	**30 mg/kg**	**60 mg/kg**
n	10	6	6	6	6	6
Post lumbar puncture syndrome	2 (20.0%)	3 (50.0%)	0	5 (83.3%)	0	2 (33.3%)
Procedural pain	1 (10.0%)	0	0	0	0	2 (33.3%)
Headache	3 (30.0%)	0	1 (16.7%)	2 (33.3%)	1 (16.7%)	5 (83.3%)
Back pain	3 (30.0%)	0	1 (16.7%)	0	1 (16.7%)	1 (16.7%)
Muscle spasms	0	0	0	0	0	2 (33.3%)
Neck pain	1 (10.0%)	0	0	0	0	2 (33.3%)
Puncture site pain	2 (20.0%)	1 (16.7%)	0	1 (16.7%)	0	1 (16.7%)
Hypercholesterolaemia	0	0	0	3 (50.0%)	0	3 (50.0%)
Nausea	2 (20.0%)	0	0	3 (50.0%)	0	1 (16.7%)
Vomiting	1 (10.0%)	0	0	2 (33.3%)	0	0
Hepatic enzyme increased	0	0	1 (16.7%)	0	2 (33.3%)	0
Hot flush	0	0	0	3 (50.0%)	0	0
Hypertension	2 (20.0%)	0	0	0	0	0

**B. FIH multiple dose**						
			**JNJ-63733657**			
	**Placebo**					
	**AD**	**HP**	**HP**	**AD**	**AD**	**HP**
	**Placebo**	**Placebo**	**5 mg/kg**	**15 mg/kg**	**30 mg/kg**	**50 mg/kg**
n	2	4	6	6	5	5
Headache	1 (50.0%)	1 (25.0%)	0	2 (33.3%)	1 (20.0%)	4 (66.7%)
Back pain	0	0	1 (16.7%)	2 (33.3%)	1 (20.0%)	1 (16.7%)
Fatigue	1 (50.0%)	1 (25.0%)	0	0	0	0
Nasopharyngitis	0	1 (25.0%)	0	1 (16.7%)	0	1 (16.7%)
Post lumbar puncture syndrome	0	0	0	1 (16.7%)	0	2 (33.3%)

**C. Japanese single dose**				
		**JNJ-63733657**		
	**Placebo**	**3 mg/kg**	**15 mg/kg**	**60 mg/kg**
n	6	6	6	6
Nasopharyngitis	0	3 (50.0%)	2 (33.3%)	1 (16.7%)
Gingivitis	0	0	2 (33.3%)	0
C-reactive protein increased	0	0	0	2 (33.3%)
Headache	1 (16.7%)	1 (16.7%)	0	0

Footnote: AD, Alzheimer’s Disease; FIH, first-in-human; HP, Healthy participants; SD, standard deviation

In Part 2 of the study, 20 (87%) of the 23 participants treated with multiple doses of JNJ-63733657 reported 1 or more AEs: 66.7% of participants treated with 5 mg/kg, 83.3% of participants treated with 15 mg/kg, 100% of participants treated with 30 mg/kg, and 100% of participants treated with 50 mg/kg. No TEAEs were reported in more than 1 participant who received JNJ-63733657 5 mg/kg. Of the 6 participants treated with placebo, 5 (83.3%) reported 1 or more AEs.

The most commonly reported TEAEs (reported by >20% of participants) by preferred term in participants who received JNJ-63733657 15 mg/kg were back pain (33.3%) and headache (33.3%). No TEAEs were reported in more than 1 participant who received JNJ-63733657 30 mg/kg. The most common TEAEs in participants who received JNJ-63733657 50 mg/kg were headache (66.7%) and post-LP syndrome (33.3%). In participants who received placebo, the most commonly reported TEAEs were headache (33.3%) and fatigue (33.3%) (Table 2B).

In the Japanese study, no serious adverse events were reported in participants receiving single doses of JNJ-63733657 (3 mg/kg–60 mg/kg), and no clinically important abnormalities were observed in vital sign parameters or laboratory values. There were no deaths or early terminations due to TEAEs. Eleven (61.1%) of the 18 participants who received JNJ-63733657 reported 1 or more AEs: 66.7% of participants treated with 3 or 60 mg/kg, and 50% of participants treated with 15 mg/kg. Of the 6 participants who received placebo, 4 participants (66.7%) reported 1 or more AEs.

The most common TEAEs (reported by >20% participants) by preferred term in participants who received JNJ-63733657 3 mg/kg was nasopharyngitis (50%). For participants who received JNJ-63733657 15 mg/kg, the most common TEAEs were nasopharyngitis (33%) and gingivitis (33%), and for participants who received JNJ-63733657 60 mg/kg, the most commonly reported TEAE was C-reactive protein increase (33%). In participants who received placebo, no TEAES were reported in more than one participant (Table 2C).

### Pharmacokinetics

**Figure 1:**
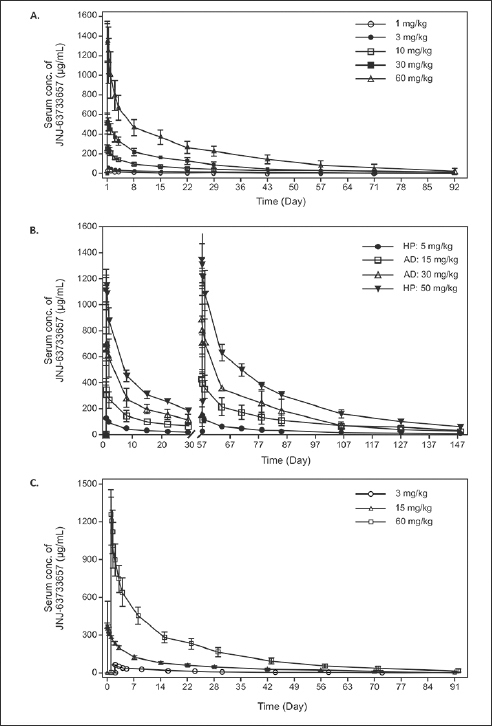
Serum concentration (mean +/− SD) time profiles of JNJ-63733657 following administration of (A) single IV doses in healthy participants in the FIH study, (B) multiple IV doses in healthy participants and participants with AD, and (C) single IV doses in healthy Japanese participants Footnote: HP, Healthy participants; AD, Alzheimer’s Disease, SD, Standard deviation

In the FIH study, linear PK in serum was observed following single IV dose administration of JNJ-63733657 from 1–60 mg/kg (Figure 1A). Median serum t_max_ ranged between 0.05 and 0.25 days after the start of administration for the 5 dose levels. Mean serum t_1/2_ ranged between 18.1 days and 26.4 days and was comparable across the 5 dose levels. Mean serum C_max_ and AUCs increased with increasing dosages, and mean values for the dose normalized serum PK parameters (C_max,dn_, AUC_last,dn_, and AUC_∞,dn_) were comparable with increasing dosages, suggesting dose proportionality in serum within the dose range evaluated (Table 3).

**Table 3:** Pharmacokinetic parameters after administration of IV doses of JNJ-63733657

	**FIH single dose**					**FIH multiple dose**^**‡**^				**Japanese single dose**		
	**1 mg/kg**	**3 mg/kg**	**10 mg/kg**	**30 mg/kg**	**60 mg/kg**	**5 mg/kg**^**§**^	**15 mg/kg**^**§**^	**30 mg/kg**^**§**^	**50 mg/kg**^**§**^	**3 mg/kg**	**15 mg/kg**	**60 mg/kg**
n	6*	5	6	6^†^	6^†^	6	6	4	6	6	6	6
C_max_, *µ*g/mL	24.6 (3.24)	62.7 (9.09)	266 (35.9)	574 (51.0)	1387 (196)	147 (26.0)	449 (69.9)	922 (265)	1348 (202)	65.6 (6.47)	374 (18.2)	1266 (186)
t_max_, day	0.11 (0.05–0.50)	0.05 (0.04–0.17)	0.25 (0.05–0.33)	0.17 (0.06–0.50)	0.17 (0.06–0.92)	0.11 (0.05–0.33)	0.17 (0.05–0.17)	0.17 (0.07–0.17)	0.07 (0.07–0.17)	0.05 (0.05–0.17)	0.06 (0.06–0.33)	0.08 (0.08–0.17)
AUC_last_, *µ*g.day/mL	322 (38.9)	819 (134)	3685 (750)	7499 (1578)	17805 (4355)	2303 (662)	8857 (3300)	15130 (5457)	24815 (2671)	775 (129)	4238 (385)	14638 (2487)
AUC_∞_, *µ*g.day/mL	339 (38.5)	849 (144)	4062 (1115)	8314 (1762)	20447 (3696)	2388 (672)	9853 (4276)	16139 (6105)	27078 (3288)	805 (148)	4399 (467)	15138 (2635)
t_l/2_, day	18.9 (3.9)	18.1 (3.0)	26.4 (7.8)	23.1 (4.6)	21.8 (6.8)	18.3 (5.3)	26.1 (6.7)	23.3 (3.4)	27.1 (2.8)	16.2 (4.0)	19.9 (3.3)	19.8 (2.4)
CL, mL/day/kg	2.97 (0.294)	3.60 (0.599)	2.58 (0.503)	3.75 (0.834)	3.03 (0.645)	3.34 (0.846)	2.87 (0.670)	3.27 (0.660)	3.23 (0.345)	3.83 (0.702)	3.44 (0.348)	4.07 (0.757)
C_max, dn_ *µ*g/mL/(mg/kg)	24.7 (3.17)	21.0 (3.15)	26.6 (3.59)	19.1 (1.70)	23.1 (3.27)	29.4 (5.20)	29.9 (4.66)	30.7 (8.83)	27.0 (4.05)	21.9 (2.16)	24.9 (1.21)	21.1 (3.10)
AUC_last, dn_ *µ*g.day/mL/(mg/kg)	322 (38.2)	274 (46.2)	369 (75.0)	250 (52.6)	297 (72.6)	461(132)	590 (220)	504 (182)	496 (53.4)	258 (43.0)	283 (25.7)	244 (41.4)
AUC_∞, dn_ µg.day/mL/(mg/kg)	340 (37.9)	284 (49.7)	406 (111)	277 (58.7)	341 (61.6)	478 (134)	657 (285)	538 (204)	542 (65.8)	268 (49.3)	293 (31.1)	252 (43.9)

* n=5 for AUC_last_, AUC_∞_, t_1/2_, CL, AUC_last, dn_ and AUC_∞, dn_; † n=5 for AUC_∞_, λz, t_1/2_, CL and AUC_∞, dn_; ‡ PK parameters calculated after 3rd dose; § 5 and 50 mg/kg administered to healthy participants; 15 and 30 mg/kg administered to participants with AD; data presented as (mean [SD], t_max_: median [range]); AUC_∞_=area under the serum concentration versus time curve from time 0 to infinity; AUClast=area under the serum concentration versus time curve from time 0 to the time corresponding to the last quantifiable serum concentration; CL=total systemic clearance; C_max_=maximum observed serum concentration of drug JNJ-63733657; _dn_=dose-normalized; IV=intravenous; n=number of subjects; SD=standard deviation; t_1/2_=apparent elimination half-life; t_max_=time to reach the maximum observed serum concentration;

Mean CSF C_max_ increased with increasing dosages. In general, the mean values for the dose normalized CSF C_max_ were comparable, and the distributions of the individual values overlapped, suggesting a dose-proportional increase of the CSF C_max_ and AUCs (data not shown). The geometric mean CSF/serum ratio on Day 2 ranged between 0.0345% and 0.0535% for all cohorts, which was lower than the observations on the other days, suggesting the equilibrium of JNJ-63733657 in CSF had not been achieved on Day 2. From Day 8 onward, the geometric mean and 95% CIs of the CSF/serum ratio ranged between 0.191% (0.115–0.319) and 0.450% (0.158–1.28) for all cohorts and was comparable for all subsequent sampling days.

In the MAD part of the study, the serum concentration-time profile showed linear PK from 5–50 mg/kg for both healthy and AD participants (Figure 1B). After administration of multiple IV doses, the median serum t_max_ ranged between 0.07 and 0.17 days after the start of the third IV administration, which generally corresponded to the sampling at the end of infusion and was consistent with the first dose. The mean serum t_1/2_ and CL were comparable for the four cohorts and similar to the SAD findings (Table 3).

The mean serum C_max_ and AUC increased with increasing dosages. Mean values for the dose normalized serum PK parameters (C_max, dn_ and AUC_τ,dn_) were comparable with increasing dosages and between healthy participants and participants with AD, suggesting dose proportionality, and were similar to the results observed following single dose administration. The mean accumulation ratio of C_max_ of the first and third dose ranged between 1.15 and 1.26 and was comparable for all cohorts. Mean accumulation ratio of AUC of the first and third dose ranged between 1.39 and 1.59 and was comparable across cohorts.

For all MAD participants, CSF concentrations were quantifiable after the first IV dose on Day 1 until the last PK sample (which varied per sampling scheme). The CSF concentrations were similar across the sampling time points within each cohort. Generally, mean maximum CSF concentrations of JNJ-63733657 increased with increasing dosages (data not shown). Following multiple doses, the geometric mean of the CSF/serum ratios were comparable for all cohorts and sampling points and were similar to those in SAD part.

In the Japanese study, serum concentration-time profiles showed linear PK after single IV doses of 3, 15 and 60 mg/kg (Figure 1C). In general, the serum PK parameters for all three dosages were comparable to those observed in non-Japanese participants (Table 3). For all three dosages evaluated in the Japanese study, quantifiable CSF concentrations were observed for all participants on day 1 after the infusion until the last CSF sample. The mean maximum CSF concentrations increased with increasing dosage. The geometric mean CSF/serum ratio on Day 2 ranged between 0.04% and 0.05% for all cohorts. From Day 14 onward, the geometric mean and 95% CIs of the CSF/serum ratio was 0.28% (0.22–0.35) for all cohorts and was comparable for all subsequent sampling days.

### Immunogenicity

Of the 53 participants receiving JNJ-63733657 in the FIH study, 11 participants (8 in SAD and 3 in MAD) were positive for treatment-emergent ADA. Six participants had a peak titer of 1:22.5, 2 had a peak titer of 1:45, 1 had a peak titer of 1:180, and 2 had a peak titer of 1:360. No treatment-emergent ADA were reported in the AD participants or participants who received placebo.

For the 2 ADA positive participants with the highest peak titers (1:360), serum JNJ-63733657 concentrations were below the lowest limit of quantification when the ADA peak titer was observed. Maximum serum concentrations were comparable for both ADA positive and ADA negative participants; however, the serum concentration-time profile of the 2 ADA positive participants with the highest peak titers showed a more rapid decline toward the end of the profile. The comparison should be interpreted with caution due to the small number of ADA-positive participants.

Of the 18 participants receiving JNJ-63733657 in the Japanese study, 1 participant in the 3 mg/kg cohort was positive for treatment-emergent ADA with peak titer of 1:22.5. The serum PK parameters for this participant did not differ from other participants in the same cohort.

### Pharmacodynamics

**Figure 2:**
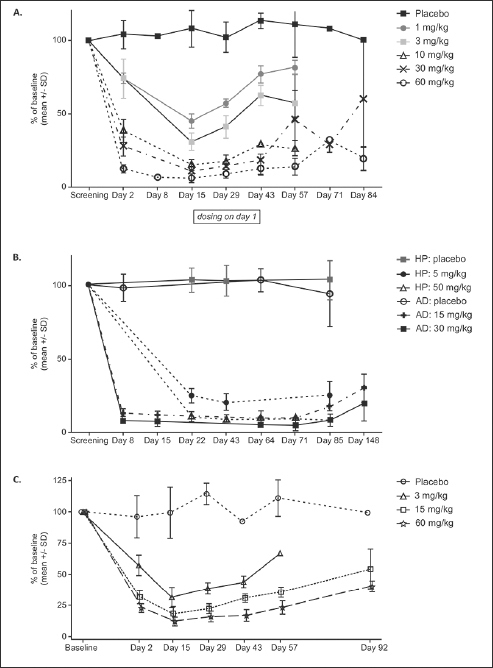
Change in CSF free p217+tau as percent of baseline (mean +/− SD) following administration of (A) Single IV doses in healthy participants in the FIH study, (B) multiple IV doses in healthy participants or participants with AD, and (C) single IV doses in healthy Japanese participants

Participants receiving single doses of JNJ-63733657 (1 mg/kg–60 mg/kg) showed dose dependent reductions in CSF free and total p217+tau, while p217+tau levels were stable in placebo-treated participants (Figure 2A and Supplementary Figure 2). The maximum reduction of CSF free p217+tau occurred at 8 days postdose, then began to rise but did not return to baseline levels by 56 days postdose. The percent of baseline total p217+tau at 14 days postdose was 105% with placebo and 54%, 42%, 30%, 32%, and 29% with 1, 3, 10, 30, and 60 mg/kg JNJ-63733657, respectively. While the baseline p217+tau concentration was variable amongst participants, all participants in each cohort exhibited a similar percent reduction in p217+tau. No change in tTau or p181tau was observed in the participants from Part 1 following single dose administration (Supplementary Figures 3 and 4).

Following multiple dose administrations of JNJ-63733657, there were dose-dependent and sustained reductions in free and total p217+tau in CSF as measured by the percentage change from baseline whereas placebo-treated participants had stable levels of p217+tau (Figure 2B). With monthly dosing, no rebound in CSF p217+tau level was observed over at least 85 days (28 days after the last dose), suggesting sustained maximal impact of JNJ-63733657. The percent of baseline total CSF p217+tau in each dose group at the final timepoint for each subject (Day 71 or 85) was: healthy participants (HP) placebo = 110%, HP 5 mg/kg = 42%, HP 50 mg/kg = 31%, AD placebo = 98%, AD 15 mg/kg = 29%, AD 30 mg/kg = 18%.

The percent reduction in free and total CSF p217+tau was relatively consistent within each cohort and sustained. The absolute change in the level of p217+tau varied based on baseline concentration (data not shown). Despite higher baseline levels of p217+tau in AD participants, the percent reduction of p217+tau by JNJ-63733657 was comparable to that seen in healthy participants.

Similar to Part 1, healthy participants in the Part 2 cohorts showed no reduction in tTau or p181tau while AD participants in Part 2 exhibited a reduction in tTau and p181tau following administration of JNJ-63733657 (Supplementary Figures 3 and 4). The percent of baseline total CSF tTau in each dose group at Day 71 or Day 85 (final timepoint for each participant) was: HP placebo = 107%, HP 5 mg/kg = 99%, HP 50 mg/kg = 98%, AD placebo = 99%, AD 15 mg/kg = 96%, AD 30 mg/kg = 78%. The percent of baseline CSF p181tau in each dose group at day 71 or day 85 was: HP placebo = 99%, HP 5 mg/kg = 99%, HP 50 mg/kg = 101%, AD placebo = 94%, AD 15 mg/kg = 96%, AD 30 mg/kg = 77%. The free, total, and bound p217+tau concentrations for participants with AD are shown in Supplementary Table 1.

Similar to the results of the FIH SAD part of the study, in the Japanese SAD study, all 18 participants who received JNJ-63733657 exhibited reductions in p217+tau (total and free) at Day 2 to Day 15 postdose, while the 6 participants who received placebo had stable levels of p217+tau (total and free). At Day 15, the mean percentage of baseline free CSF p217+tau was 31.36%, 18.28%, and 12.22%, and the total CSF p217+tau was 49.97%, 35.61%, and 35.18% for JNJ-637336573 3, 15, and 60 mg/kg groups, respectively (Figure 2C). While dose dependent reductions of p217+tau in CSF were observed, there were no changes in tTau or p181tau in healthy Japanese participants following administration of JNJ-6373365.

## Discussion

These Phase 1 randomized, double-blind, placebo controlled, single and multiple dose studies evaluated the safety and tolerability, PK, immunogenicity, and PD of the anti-tau monoclonal antibody JNJ-63733657 in healthy participants and participants with prodromal or mild AD. JNJ-63733657 was generally safe and well-tolerated, demonstrated linear and dose-proportional PK following single and multiple doses, and showed dose dependent decreases in levels of free p217+tau in the CSF. The PK and PD profiles were similar in the healthy participants and participants with AD, and sustained suppression in free p217+tau was observed over at least 85 days (28 days after the last dose) following multiple dosing.

From a safety perspective, JNJ-63733657 was generally safe and well tolerated. No safety concerns were identified following single dose administration (1 mg/kg–60 mg/kg, IV) in healthy participants (non-Japanese and Japanese) or following multiple dose administration (monthly × 3 doses, IV) in healthy participants (5 mg/kg or 50 mg/kg) or participants with prodromal or mild AD (15 mg/kg or 30 mg/kg). The number of AEs associated with lumbar punctures was higher than might be expected and may be attributable in part to smaller size, atraumatic spinal needles not being utilized by all sites. Of note, there were no clinically important changes in the CSF protein or cell counts to suggest an inflammatory process.

In the FIH study, the observed PK parameters are in line with other monoclonal antibodies with an observed t_1/2_ ranging from 18.3–27.1 days, CSF/serum ratio ∼ 0.2% after single and multiple doses, and the PK profiles were similar in healthy participants and participants with AD. Following single and multiple dose administration, JNJ-63733657 showed linear PK across all tested dose levels. The dose-normalized serum PK parameters were comparable between 1–60 mg/kg SAD and 5–50 mg/kg MAD, suggesting dose proportionality. The accumulation ratio was found to be 1.15–1.26 for C_max_ and 1.39–1.59 for AUC_t_, suggesting limited accumulation after monthly infusions. The PK profiles in Japanese and non-Japanese participants following single doses of JNJ-63733657 were similar. Treatment emergent ADAs were detected in a subset of participants, with the incidence being greater at later timepoints. For the 3 participants with the highest peak titers (1:180–1:360), the serum concentration of JNJ-63733657 was BLQ at later timepoints, indicating the high peak ADA titers may be associated with faster clearance of JNJ-63733657; however, this observation should be interpreted with caution due to the small number of ADA-positive participants.

The reduction in levels of free p217+tau in the CSF demonstrated in this study suggests engagement of the antibody with the epitope of interest on extracellular tau, which might reflect changes in the levels of extracellular tau seeds containing the p217+ epitope in the ISF. Although extracellular p217+tau species are mainly monomeric, potential multimeric species also are expected to be targeted by the antibody. Such interception of tau seeds may attenuate or halt the progression of tauopathy. Importantly, total p217+tau levels in the CSF also were reduced by JNJ-63733657 suggesting that, in addition to epitope binding, active clearance may also occur. Such a mechanism has also been observed in the clinical study of the IgG4 tau monoclonal antibody semorinemab, where modest drug-dependent declines in CSF total tau also were reported (28). The p217+tau reductions in the current study may be due to clearance by microglia, which is a proposed mechanism of action for tau IgG1 monoclonal antibodies (29). To further support this hypothesized mechanism of action, the lower levels of p217+tau in the CSF were confirmed via liquid chromatography mass spectroscopy (30) confirming removal of tau peptides when bound to the antibody. Interestingly, no change in tTau or p181tau was observed in healthy participants following single dose administration, which can be attributed to the specificity of JNJ-63733657 for p217+tau and the low percent of tTau that contains the p217 epitope (<5%) (31) in healthy participants. The reductions of tTau and p181tau in the AD participants and not healthy participants may be due to a higher percentage of total tau species being phosphorylated at the p217 epitope in AD participants (8.5%) (31) as compared with healthy participants (1.9%) (30). As such, an impact on p217+tau would be more evident in the tTau and p181tau measurements in AD participants. Additionally, the reduction in tTau in participants with AD also may reflect clearance of antibody-p217+tau complexes by microglia in the CNS.

Following both single dose or multiple dose administration, the percent reduction in p217+tau was similar within a given dose cohort regardless of the baseline absolute tau level. Moreover, in AD participants, where the baseline p217+tau levels were higher than in healthy participants, the percent reduction was comparable to that seen in healthy participants. These observations suggest a dynamic equilibrium.

There are several characteristics of JNJ-63733657 that are of interest. While other anti-tau mAbs have failed to show an effect on disease progression in AD (22, 23), JNJ-63733657 differs from these other antibodies in that it binds to the proline rich domain in the mid-region of tau rather than the N-terminus. It is known that there is cleavage of tau at the N-terminus, and thus antibodies targeting this region may be less effective in neutralizing tau seeds due to truncation. Moreover, JNJ-63733657 targets the p217+ epitope. Phosphorylated tau, especially at amino acid 217, has emerged as a biomarker associated with brain amyloid and tau burden supporting its relevance to the disease (32, 33).

While targeting p217+tau may have therapeutic potential, there are caveats to consider. As mentioned previously, it is not possible to directly measure the true target, tau seeds, and decreases in p217+tau in the CSF can be viewed only as a potential indirect indicator of the impact on the tau seed. Additionally, the potential efficacy of JNJ-63733657 is predicated on the hypothesis that tau seeds spread in a prion like manner via the ISF. However, the mechanism of tau spread is not known; it is possible that alternative mechanisms such as tunneling nanotubes or exosomes may be relevant (12), in which case tau seeds would evade the ISF where the antibody is presumed to act. It currently is not possible to directly measure the tau seed in the ISF to better understand the mechanism of spread.

## Conclusions

Single and multiple dose administration of JNJ-63733657 demonstrated dose dependent, sustained reductions in CSF p217+tau levels. The clinical and pharmacokinetic profile as well as the p217+tau biomarker response observed in these Phase 1 trials are favorable and support further study of JNJ-63733657 to determine if this antibody is able to slow tau spread and the clinical course of AD. A Phase 2 study evaluating JNJ-63733657 in prodromal AD or mild AD dementia is currently ongoing (NCT04619420)(34).

## Electronic supplementary material

Supplementary material, approximately 357 KB.

Supplementary material, approximately 28.9 KB.
