# Supplementary material for: Phase 1 Studies of the Anti-Tau Monoclonal Antibody JNJ-63733657 in Healthy Participants and Participants with Alzheimer’s Disease
Source: J Prev Alzheimers Dis. 2024 Sep 10;11(6):1592–603. doi: 10.14283/jpad.2024.163 (PMC11573813; doi:10.14283/jpad.2024.163)
Supplement: Supplementary file 1 — Supplementary material, approximately 357 KB. [file 42414_2024_365_MOESM1_ESM.docx]

**Supplementary Figure 1:** Pharmacodynamic effects of JNJ-63733657 in CSF: Measurements of free p217+tau and total p217+tau, allowing determination of p217+tau bound to JNJ‑63733657.

1.
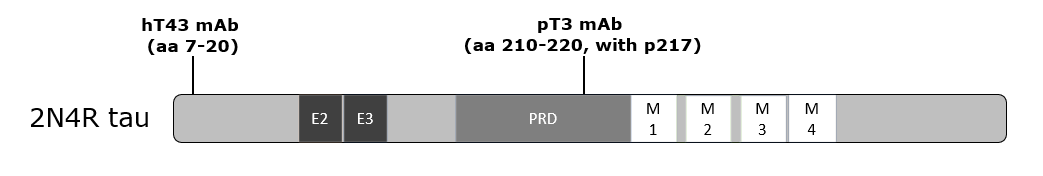
Tau molecule indicating epitopes of the Simoa assay reagents. The CSF p217+tau assay uses the murine version of JNJ-63733657 (pT3) as the capture antibody which recognizes aa 210-220 with obligatory phosphorylation at 217 and enhanced binding when neighboring epitopes are phosphorylated. The detection antibody (hT43) recognizes amino acids 7-20. The assay signal is reduced when JNJ-63733657 is present due to competition for epitope binding (27).

27. Triana-Baltzer G, Van Kolen K, Theunis C, et al. Development and Validation of a High Sensitivity Assay for Measuring p217 + tau in Cerebrospinal Fluid. J Alzheimers Dis 2020; 77:1417-1430

1. Tau is a heat stable molecule, while antibodies are not. Therefore, heat denaturation allows for measurement of all p217+tau molecules in the presence of binding antibodies. FREE p217+tau (left column) is measured in non-denatured CSF and quantifies p217+tau not bound by JNJ-63733657. TOTAL p217+tau (middle column) is measured after heat denaturation of the CSF and quantifies all p217+tau (FREE + BOUND). After denaturation p217+tau can be bound by the pT3-coated Simoa beads, which are added after the heat denaturation step. BOUND p217+tau (right column) reflects TOTAL minus FREE p217+ tau, and thus quantifies p217+tau bound by JNJ-63733657.


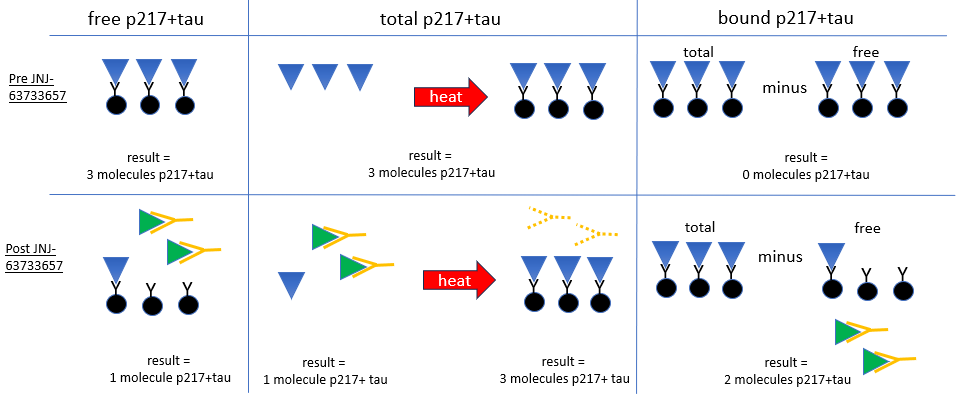


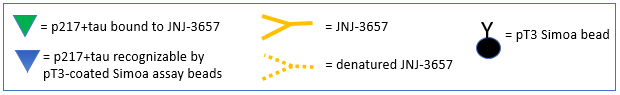


C. CSF spiked with JNJ-63733657 (1 ug/ml) in vitro demonstrates competition of Simoa assay signal (1st 2 bars). Heat denaturation of the CSF (≥2 minutes), rescues the Simoa assay signal (last 6 bars)

**Supplementary Figure 2**. Change in CSF total p217+tau as percent of baseline (mean +/- SD) following administration of (A) Single IV doses in healthy participants in the FIH study, and (B) multiple IV doses in healthy participants or participants with AD. The Japanese study showed similar results (data not shown).

A.


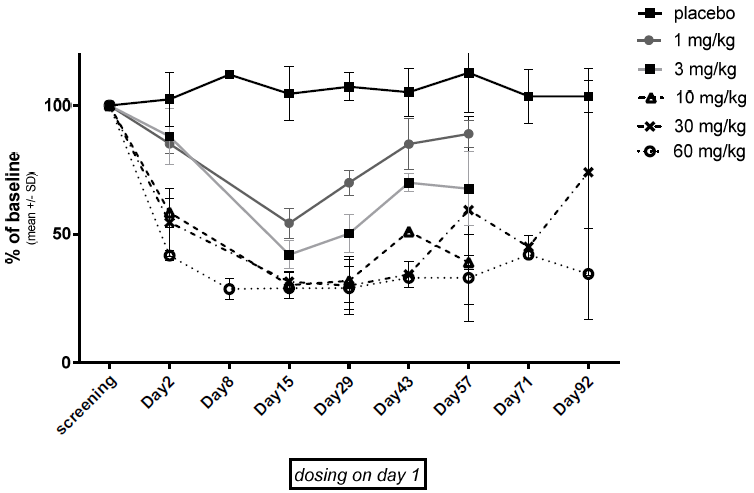


**B.**


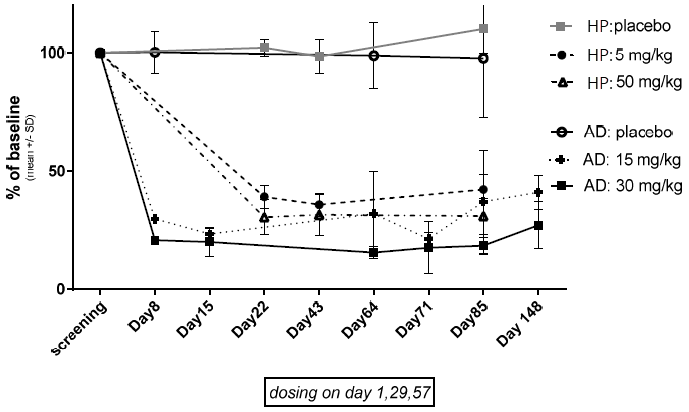


Footnote: HP, Healthy participants; AD, Alzheimer’s Disease; SD, Standard deviation

**Supplementary Figure 3**. Change in CSF total tau (tTau) as percent of baseline (mean +/- SD) following administration of (A) Single IV doses in healthy participants in the FIH study, and (B) multiple IV doses in healthy participants or participants with AD. The Japanese study showed similar results (data not shown).

A.


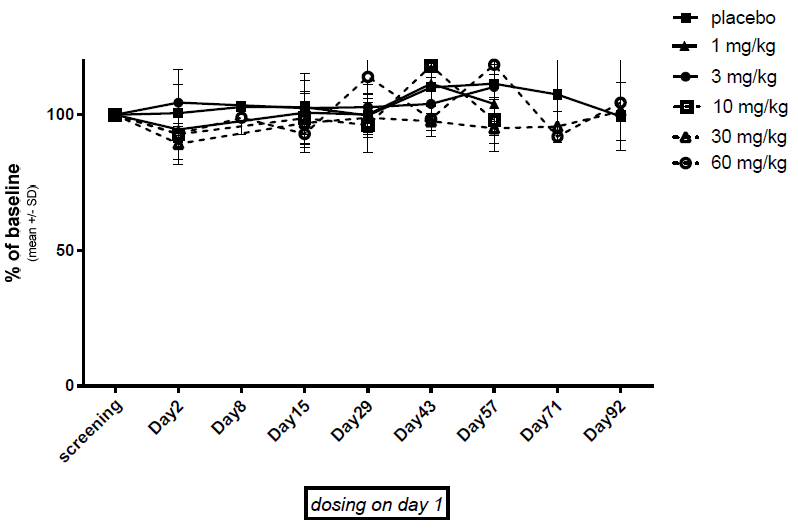


B.


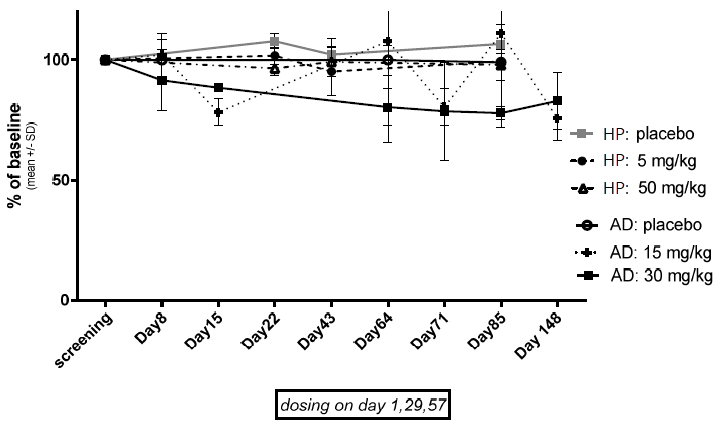


Footnote: HP, Healthy participants; AD, Alzheimer’s Disease; SD, Standard deviation

**Supplementary Figure 4**. Change in CSF p181tau as percent of baseline (mean +/- SD) following administration of (A) Single IV doses in healthy participants in the FIH study, and (B) multiple IV doses in healthy participants or participants with AD. The Japanese study showed similar results (data not shown).

A.


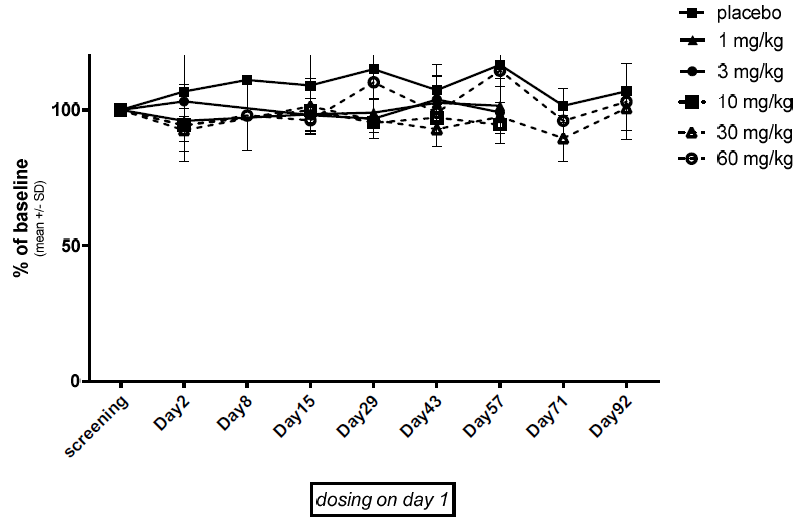


B.


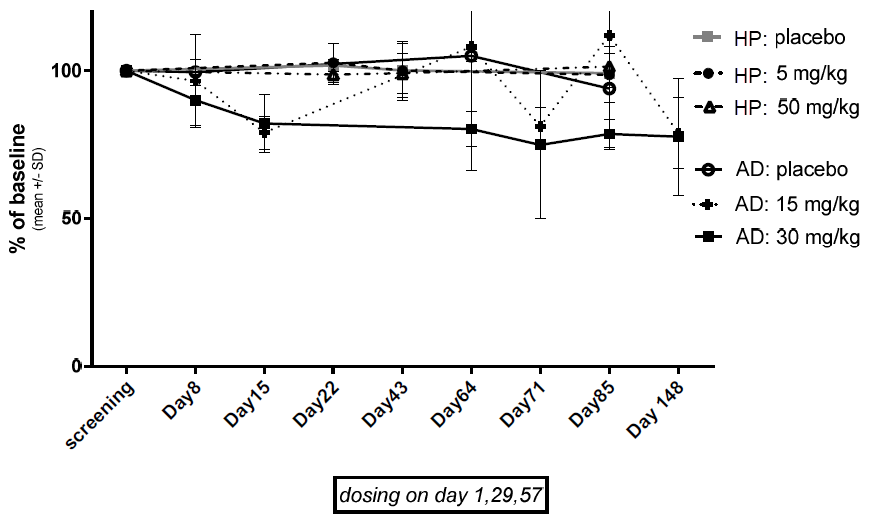


Footnote: HP, Healthy participants; AD, Alzheimer’s Disease; SD, Standard deviation
