# Supplementary material for: Phase 1 Studies of the Anti-Tau Monoclonal Antibody JNJ-63733657 in Healthy Participants and Participants with Alzheimer’s Disease
Source: J Prev Alzheimers Dis. 2024 Sep 10;11(6):1592–603. doi: 10.14283/jpad.2024.163 (PMC11573813; doi:10.14283/jpad.2024.163)
Supplement: Supplementary file 2 — Supplementary material, approximately 28.9 KB. [file 42414_2024_365_MOESM2_ESM.docx]

**Supplemental Table 1**

**A- Individual CSF Concentrations (Absolute Values) of p217+tau (total, bound and free) following multiple doses of 15 mg/kg JNJ-63733657 administered intravenously on Day 1, Day 29 and Day 57** **in participants with prodromal or mild AD.**

Bound is calculated as the difference between total and free p217+tau.

|  |  | |  | | | **CSF concentrations of p217+tau (total) (pmol/L)** | | | | | | | | | | | | |  |
| --- | --- | --- | --- | --- | --- | --- | --- | --- | --- | --- | --- | --- | --- | --- | --- | --- | --- | --- | --- |
| **Subject** | **Screening** | | **Day 8** | | | **Day 15** | | **Day 64** | | **Day 71** | | | | **Day 85** | | **Day 148** | | |  |
| 1^a^ | 2.0104 | | 0.6196 | | | - | | 1.0247 | | - | | | | 1.2245 | | - | | |  |
| 2^b^ | 4.8471 | | - | | | 1.0207 | | - | | 0.9415 | | | | - | | 2.2415 | | |  |
| 3^a^ | 1.3807 | | 0.398 | | | - | | 0.4099 | | - | | | | 0.4405 | | - | | |  |
| 4^a^ | 9.6624 | | 2.8901 | | | - | | 1.4753 | | - | | | | 1.79 | | - | | |  |
| 5^b^ | 9.1568 | | - | | | 2.1653 | | - | | 1.8444 | | | | - | | 3.2878 | | |  |
| 6^b^ | 3.5872 | | - | | | 0.9227 | | - | | 0.8791 | | | | - | | NS | | |  |
| **Subject** | | **CSF concentrations of p217+tau (free) (pmol/L)** | | | | | | | | | | | | | | | | | |
|  |  | **Screening** | | **Day 8** | | | **Day 15** | | **Day 64** | | | **Day 71** | | | **Day 85** | | | **Day 148** | |
| 1^a^ | | 2.1179 | | 0.2921 | | | - | | 0.3159 | | | - | | | 0.5806 | | | - | |
| 2^b^ | | 4.8192 | | - | | | 0.6061 | | - | | | 0.4589 | | | - | | | 1.7691 | |
| 3^a^ | | 1.4601 | | 0.1627 | | | - | | 0.1395 | | | - | | | 0.2004 | | | - | |
| 4^a^ | | 10.1193 | | 1.5767 | | | - | | 0.6609 | | | - | | | 1.1123 | | | - | |
| 5^b^ | | 10.5903 | | - | | | 1.104 | | - | | | 0.8573 | | | - | | | 2.5095 | |
| 6^b^ | | 4.0731 | | - | | | 0.568 | | - | | | 0.4447 | | | - | | | NS | |
| **Subject** | **CSF concentrations of p217+tau (bound) (pmol/L)** | | | | | | | | | | | | | | | | | |  |
|  | **Screening** | | | | **Day 8** | | **Day 15** | **Day 64** | | | **Day 71** | | **Day 85** | | | | **Day 148** | |  |
| 1^a^ | 0 | | | | 0.328 | | - | 0.709 | | | - | | 0.644 | | | | - | |  |
| 2^b^ | 0.0279 | | | | - | | 0.415 | - | | | 0.483 | | - | | | | 0.472 | |  |
| 3^a^ | 0 | | | | 0.235 | | - | 0.270 | | | - | | 0.240 | | | | - | |  |
| 4^a^ | 0 | | | | 1.31 | | - | 0.814 | | | - | | 0.678 | | | | - | |  |
| 5^b^ | 0 | | | | - | | 1.06 | - | | | 0.987 | | - | | | | 0.778 | |  |
| 6^b^ | 0 | | | | - | | 0.355 | - | | | 0.434 | | - | | | | NS | |  |

-: Timepoint not included in CSF sampling scheme.

NS: No Sample.

a Subject randomized to CSF sampling scheme 1: Baseline, Day 8, Day 64 and Day 85.

b Subject randomized to CSF sampling scheme 2: Baseline, Day 15, Day 71 and Day 148.

**B. Individual CSF Concentrations (Absolute Values) of p217+tau (total, bound and free) following multiple doses of 30 mg/kg JNJ-63733657 administered intravenously on Day 1, Day 29 and Day 57 in participants with prodromal or mild AD.**

Bound is calculated as the difference between total and free p217+tau.

| **Subject** | **CSF concentrations of p217+tau (total) (pmol/L)** | | | | | | | | | | |
| --- | --- | --- | --- | --- | --- | --- | --- | --- | --- | --- | --- |
|  | **Screening** | **Day 8** | | | **Day 15** | **Day 64** | **Day 71** | | | **Day 85** | **Day 148** |
| 7^c^ | 14.5168 | NS | | | NS | NS | NS | | | NS | NS |
| 8^b^ | 8.2609 | - | | | 2.0124 | - | 2.1062 | | | - | 2.8219 |
| 9^a^ | 11.0875 | 2.3983 | | | - | 1.5265 | - | | | 1.7692 | - |
| 10^b^ | 5.563 | - | | | 0.8815 | - | 0.5489 | | | - | 1.1235 |
| 11^a^ | 8.7482 | 1.752 | | | - | 1.5322 | - | | | 1.8448 | - |
| **Subject** | **CSF concentrations of p217+tau (free) (pmol/L)** | | | | | | | | | | |
|  | **Screening** | **Day 8** | | | **Day 15** | **Day 64** | **Day 71** | | | **Day 85** | **Day 148** |
| 7^c^ | 14.4497 | NS | | | NS | NS | NS | | | NS | NS |
| 8^b^ | 7.001 | - | | | 0.7049 | - | 0.5336 | | | - | 1.9291 |
| 9^a^ | 11.8096 | 0.9147 | | | - | 0.4785 | - | | | 0.6516 | - |
| 10^b^ | 6.1407 | - | | | 0.3378 | - | 0.1562 | | | - | 0.6824 |
| 11^a^ | 8.1192 | 0.6574 | | | - | 0.5322 | - | | | 0.9751 | - |
| **Subject** | **CSF concentrations of p217+tau (bound) (pmol/L)** | | | | | | | | | | |
|  | **Screening** | | **Day 8** | **Day 15** | | **Day 64** | | **Day 71** | **Day 85** | | **Day 148** |
| 7^c^ | 0.0671 | | NS | NS | | NS | | NS | NS | | NS |
| 8^b^ | 1.26 | | - | 1.31 | | - | | 1.57 | - | | 0.893 |
| 9^a^ | 0 | | 1.48 | - | | 1.05 | | - | 1.12 | | - |
| 10^b^ | 0 | | - | 0.544 | | - | | 0.393 | - | | 0.441 |
| 11^a^ | 0.629 | | 1.09 | - | | 1.00 | | - | 0.870 | | - |

-: Timepoint not included in CSF sampling scheme.

NS: No Sample.

a Subject randomized to CSF sampling scheme 1: Baseline, Day 8, Day 64 and Day 85.

b Subject randomized to CSF sampling scheme 2: Baseline, Day 15, Day 71 and Day 148.

c CSF sampling scheme unknown due to multiple missing samples.
